# Associations of Existing Diabetes, Gestational Diabetes, and Glycosuria with Offspring IQ and Educational Attainment: The Avon Longitudinal Study of Parents and Children

**DOI:** 10.1155/2012/963735

**Published:** 2012-08-13

**Authors:** Abigail Fraser, Scott M. Nelson, Corrie Macdonald-Wallis, Debbie A. Lawlor

**Affiliations:** ^1^MRC Centre for Causal Analyses in Translational Epidemiology, School of Social & Community Medicine, University of Bristol, Bristol BS8 2BN, UK; ^2^School of Medicine, University of Glasgow, Glasgow G11 6NT, UK

## Abstract

*Introduction*. Results from studies examining associations of maternal diabetes in pregnancy with offspring cognitive outcomes have been inconclusive. *Methods*. We used data from the Avon Longitudinal Study of Parents and Children, a UK prospective pregnancy cohort. Outcomes were School Entry Assessment (SEA) scores (age 4, *N* = 6, 032) and WISC-III IQ (age 8, *N* = 5, 282–5,307) and General Certificate of Secondary Education (GCSE) results (age 16, *N* = 7, 615). *Results*. Existing diabetes, gestational diabetes, and, to a lesser extent, glycosuria were associated with lower offspring SEA scores (age 4), IQ (age 8), and GCSE results (age 16) even when adjusting for offspring sex, maternal age, prepregnancy BMI, smoking in pregnancy, parity, caesarean section, maternal education, and occupational social class. Offspring of mothers with existing diabetes had a threefold risk of achieving no GCSEs graded A*-C, whilst offspring of women with gestational diabetes had, on average, a five point lower IQ compared to offspring of women with no diabetes or glycosuria. *Conclusions*. Maternal diabetes in pregnancy is consistently associated with lower offspring cognition and educational attainment though confidence intervals were wide. The weaker associations with glycosuria suggest a dose-dependent adverse association with IQ.

## 1. Introduction

Maternal diabetes is associated with neonatal complications that may in turn adversely affect psychomotor development [1, 2]. Maternal diabetes may also directly affect fetal neurodevelopment due to changes in brain structure via in-utero exposure to a metabolic milieu which incorporates high or fluctuating concentrations of glucose and potentially ketonemia [3]. The overall impact on cognitive function of this altered metabolic environment is contentious as ketosis adversely affects neurodevelopment [4], while increased concentrations of glucose may be beneficial [5]. 

Several studies have examined associations of maternal diabetes and metabolic indices in pregnancy with offspring cognitive function. Most of these have found inverse or no associations between maternal diabetes in pregnancy and various cognitive outcomes [2, 6–10]. In the largest study to date [11], maternal diabetes in pregnancy was associated with, worse educational attainment at the age of 16. However, this study could not distinguish between maternal type 1, type 2, and gestational diabetes. Inverse associations between the degree of maternal metabolic control and cognitive outcomes have also been reported [2, 12]. In contrast, in a recent report from India, gestational diabetes was associated with higher cognitive scores in offspring [5]. 

Here we report on the associations of maternal diabetes/glycosuria with several cognitive outcomes in offspring measured throughout childhood and adolescence in a contemporary birth cohort. We studied cognitive outcomes and educational attainment at different ages to assess whether potential association was changed with age as well as to ascertain any practical implications for achievements later on in life.

## 2. Methods

**Figure 1:**
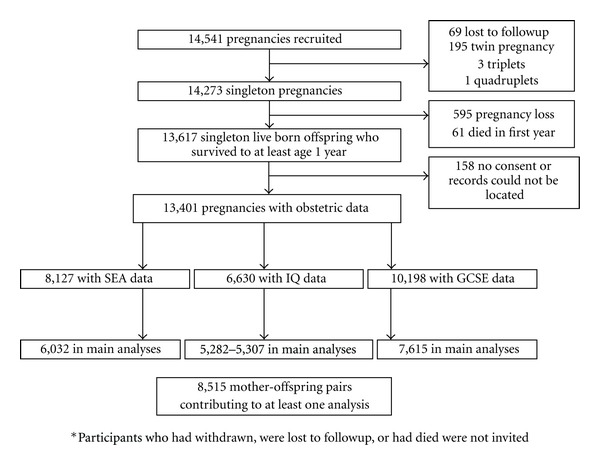
Participant flow chart.

The Avon Longitudinal Study of Parents and Children (ALSPAC) is a prospective population-based birth cohort study that recruited some 14,541 pregnancies resident in Avon, UK with expected dates of delivery April 1st 1991 to December 31st 1992 (http://www.alspac.bris.ac.uk/). 13,617 singleton offspring survived to at least one year of age.  Figure 1 shows the participant flow chart for this study.

Ethical approval was awarded by the ALSPAC Law and Ethics Committee and the Local Research Ethics Committee [13, 14].

### 2.1. Pregnancy Diabetes

Information on existing maternal diabetes and past history of gestational diabetes was collected by questionnaire from women at the time of recruitment. A standard protocol was used by research midwives to obtain information on gestational diabetes and glycosuria (recorded as none, trace, +, ++, +++, or more) for the index pregnancy from the woman's antenatal and postnatal medical records. The practice in the UK at the time was for all women to be offered urine tests for glycosuria at each antenatal clinic visit. Universal screening of women with a random or fasting blood glucose level or with an oral glucose tolerance test was not undertaken, and diagnostic tests for gestational diabetes will only have been undertaken in women with established risk factors (family history, previous history of gestational diabetes or macrosomic birth, South Asian ethnicity) or persistent glycosuria. Glycosuria was defined as a record of at least ++ (equal to 13.9 mmoL/L or 250 mg/100 mL according to the manufacturer (Bayer)) on at least two occasions at any time during the pregnancy [15]. Women were classified into one of four mutually exclusive categories: no evidence of glycosuria or diabetes; existing diabetes before the pregnancy; gestational diabetes; glycosuria.

### 2.2. Cognitive Outcomes

We used cognitive measures obtained at three different ages: School Entry Assessment (SEA) results (age 4), IQ (age 8), and General Certificate of Secondary Education (GCSE) results (age 16). SEA is assessed for every UK child beginning school education. Here, we use a summary of the results for the four required skills (language, reading, writing, and mathematics) ranging from 0 to 20. At 8 years, cognitive function was measured by Wechsler Intelligence Scale for Children (WISC-III) as part of the 8 year follow-up examination [16]. The WISC–III comprises 10 subtests (5 verbal and 5 performance subtests) that sum to the verbal IQ and performance IQ and to produce a full-scale IQ. Scores were age standardized according to the WISC manual. Children at state secondary school are mandatorily required to study English, Mathematics, and Science with this formally assessed by the GCSE examination at age 16. Conventional practice is to study 10 GCSE subjects. We encoded GCSE results as 2 binary measures a priori: a measurement of 5 or more GCSEs at grades A*-C including Mathematics and English (this measurement is of relevance as it is the requirement to continue into higher/further education and for many semiskilled jobs) and a measurement of 0 GCSEs grades A*-C, as a measure of low achievement. SEA and GCSE results were obtained by record linkage to the National Pupil Database [13].

### 2.3. Other Variables

Maternal age, mode of delivery (caesarean section/vaginal delivery), and the child's sex were obtained from the obstetric records. Information on parity and maternal smoking in pregnancy (grouped as 1 (never smoked), 2 (smoked before pregnancy or in first trimester then stopped), and 3 (smoked throughout pregnancy)), height and prepregnancy weight as well as hypertension prior to pregnancy were obtained from questionnaire responses. On the basis of questionnaire responses—the highest parental occupation was used to allocate the children to family social class groups (classes I (professional/managerial) to V (unskilled/manual workers)); maternal education was categorised as university level yes/no. Gestational age and infant birthweight were recorded in the delivery room and abstracted from obstetric records and/or birth notifications. We computed birthweight standardized for gestational age. Duration of breast feeding (assessed by questionnaire at 15 months) was categorised as never, 0–3 months, 3–5 months, and 6+ months.

### 2.4. Statistical Analysis

The distribution of characteristics across the different categories of pregnancy diabetes was examined using univariable linear and logistic regression models as appropriate. Multivariable linear (SEA and IQ) or logistic regression (GCSE results) was used to examine the associations of existing diabetes, gestational diabetes and glycosuria versus no diabetes or glycosuria with each outcome. In the basic model, we adjusted for maternal age at birth and offspring sex. In the second model, we additionally adjusted for potential confounding by prepregnancy BMI, maternal smoking in pregnancy, parity, mode of delivery, maternal education and occupational social class. Finally, in model 3 we also added terms for birthweight standardized for gestational age, gestational age and duration of breast feeding as potential mediators. Model 3 includes a smaller sample size due to missing data on birthweight and duration of breast feeding. 145 sibships were included in our sample. We repeated all analyses excluding the younger sibling so that each woman was included only once. Results were unchanged from those presented here.

### 2.5. Missing Data

We undertook a sensitivity analysis aimed at exploring whether missing covariable data might have biased our results. We imputed missing covariable data and also conducted analyses on the 8,127 mother-offspring pairs who had complete data on complications in pregnancy and School Entry Assessment scores; 6,630 mother-offspring pairs who had complete data on complications in pregnancy and WISC assessed IQ, and 10,198 mother-offspring pairs who had complete data on complications in pregnancy and GCSEs (see Figure 1). We used multivariable multiple imputation in Stata as described by Royston [17]. We carried out 20 cycles of regression switching and generated 20 imputation datasets. The multiple multivariate imputation approach creates a number of copies of the data in which missing values are imputed by chained equations [17]. Results are obtained by averaging across the separate results from each of these datasets using Rubin's rules, and the procedure takes account of uncertainty in the imputation so that the standard errors for any regression coefficients (used to calculate *P* values and 95% confidence intervals) take account of uncertainty in the imputations as well as uncertainty in the estimation [17].

## 3. Results

Of the 8,515 women contributing to at least one of the analyses, 26 (0.3%) had diabetes before pregnancy, 33 (0.4%) had gestational diabetes, and 264 (3.1%) had glycosuria. Of the 26 women with preexisting diabetes, all were diagnosed before 29 years of age, 19 were treated with insulin, 2 with other drugs, and 4 with diet only. Results did not differ when removing the four mothers treated by diet only.  Table 1 presents the characteristics of mothers and offspring included in the analyses across categories of no pregnancy diabetes/glycosuria, preexisting diabetes, gestational diabetes, and glycosuria. Differences between these were found for prepregnancy BMI with women with gestational diabetes having the highest mean BMI, age at delivery; birthweight, the proportion of male offspring, and total and verbal IQ.

Table 2 presents associations of preexisting diabetes, gestational diabetes, and glycosuria with outcomes. In model 1, preexisting diabetes, gestational diabetes, and glycosuria were all associated with lower SEA scores. The point estimate for preexisting diabetes was the largest in magnitude, but the association with glycosuria was the only one to reach statistical significance, likely due to the larger number of women in this category. Adjustment for confounders (model 2) resulted in marked attenuation of the association of gestational diabetes with SEA, with a lesser effect on the glycosuria estimate and none on the estimate for preexisting diabetes. Further adjustment for potential mediators (model 3) resulted in a marked attenuation of the association of preexisting diabetes with offspring SEA and in a change in the direction of association for gestational diabetes, though confidence intervals spanned the null, whilst the inverse association of glycosuria with SEA scores remained.

All three categories of pregnancy diabetes were inversely associated with offspring total IQ, with the largest point estimates observed for gestational diabetes (models 1-2). All confidence intervals included the null except that for gestational diabetes in model 1. Adjusting for confounders attenuated associations toward the null (model 2), whilst in model 3 the association for preexisting diabetes was greatly attenuated, but for gestational diabetes and glycosuria, the point estimate increased in magnitude. Associations with verbal IQ were similar to those with total IQ, with inverse associations observed for all categories of pregnancy diabetes, and the strongest being for gestational diabetes (models 1–3). Inverse associations were also observed for performance IQ however as the magnitude of the point estimates were greater for preexisting diabetes than for gestational diabetes and all confidence intervals included the null. Preexisting diabetes, gestational diabetes, and glycosuria were associated with a lower odds of scoring A*-C on 5+ GCSEs (models 1–3), but again, confidence intervals included the null in all models. Offspring of women with preexisting diabetes and, to a lesser extent, women with gestational diabetes, were at increased risk of achieving no A*-C GCSEs, whilst no association, was observed for glycosuria (model 1 and 2). Adjusting for potential mediators only strengthened the observed associations but for gestational diabetes the confidence interval still included the null.

Results of the sensitivity analysis using the imputed datasets are presented in Table 3 and were essentially the same as those presented in Table 2 but with more precision (narrower confidence intervals). Of note, offspring of women with preexisting diabetes did worse than offspring of women with gestational diabetes in relation to total and verbal IQ and no A*-C GCSEs. In addition, in the analyses using imputed datasets, adjusting for mediators in model 3 (for which there was the greatest amount of missing data) did not substantially affect the point estimates from the confounder adjusted model (model 2).

## 4. Discussion

In this contemporary birth cohort, maternal impaired glycaemic status in pregnancy was associated with lower School Entry Assessment score (age 4), IQ (age 8), and educational attainment (age 16). The overwhelming majority of point estimates consistently suggested that outcomes were worse in offspring of women with impaired glycemic status in pregnancy. Although due to limited numbers of women with impaired glycaemic status, confidence intervals were relatively wide. The suggested impact on educational attainment at 16 is particularly important given that it has significant implications for progression to higher education, future employment prospects, and hence income. 

Our observed adverse association of preexisting maternal diabetes on long-term cognition is consistent with previously reported associations between maternal diabetes and worse school achievement at 16 years of age, although the authors in that study were unable to examine the relative contribution of preexisting and gestational diabetes [11]. In contrast, exposure to gestational diabetes was primarily associated with IQ scores, but the overall effect on national curriculum achievement at age 16 was less pronounced. It is possible that this difference in relation to school achievement at 16 is due to type 1 diabetes being a lifetime disease requiring monitoring and careful management which may have practical adverse implications for offspring schooling in terms of maternal support. The associations of gestational diabetes and indeed of preexisting diabetes with offspring IQ may reflect a true *intrauterine* mechanism, but results need replication in larger studies that can also address causality, for example, by comparing cognitive development in siblings discordant in their exposure to maternal gestational diabetes. The weaker but similar direction associations with isolated glycosuria are striking and are in keeping with previous analyses suggesting a continuum of risk for maternal glycaemic status on offspring outcomes [15, 18]. However, we cannot exclude the possibility that this category includes women with gestational diabetes since universal screening for gestational diabetes is not standard clinical practice in the UK [19]. 

### 4.1. Limitations

The main study limitation is the small number of women with diabetes. A sensitivity analysis in which missing covariable data was imputed to increase the study power essentially yielded the same results as those presented with narrower confidence intervals. Whilst this approach does not address missing outcome data, our results would be biased only if associations were different amongst those excluded from analyses because of missing outcome data, and there is no reason to believe that this is the case. As noted previously, an additional limitation is that we cannot exclude the possibility that women with gestational diabetes were misclassified as having glycosuria due to the lack of universal screening for gestational diabetes. While this would mean that associations for glycosuria were biased away from the null, it does not affect our overall findings of an association between impaired maternal glycemia in pregnancy and offspring cognitive outcomes. Moreover, results are relevant as universal screening for gestational diabetes is not currently practiced in the UK. As per previous studies [11], we were unable to differentiate whether the preexisting diabetes was type I or type II. We presume a large proportion of the 19 managed with insulin were type 1, based on their age at diagnosis and the expected prevalence of type 1 diabetes (1 in 250), suggesting that there should be approximately 34 individuals with type 1 diabetes in the cohort. The lack of information on maternal metabolic control during pregnancy is a further limitation. Hence, larger studies with more detailed measures of maternal glycaemic and metabolic status and with repeat measures of the same cognitive tests at different ages may be valuable in shedding further light on potential mechanisms.

In summary, gestational diabetes, preexisting diabetes and, to a lesser extent, glycosuria in pregnancy were consistently associated with worse offspring school entry assessment scores, IQ and GCSE scores, though confidence intervals were wide. Results suggest that the intra uterine environment may have long term effects on offspring cognition.

## Figures and Tables

**Table 1:** Characteristics (mean (SD) or %) of mothers and offspring included in at least one analysis *N* = 8,515.

Characteristic	No diabetes/Glycosuria	Preexisting diabetes	Gestational diabetes	Glycosuria	*P* value for difference between groups
	(*N* = 8,192)	(*N* = 26)	(*N* = 33)	(*N* = 264)	
Maternal					
Manual social class, %	17.7	19.2	21.2	22.4	0.28
University education, %	13.7	15.4	9.1	9.5	0.18
Prepregnancy BMI, kg/m^2^	22.9 (3.8)	24.3 (4.5)	25.7 (5.7)	23.7 (4.3)	<0.001
Age at delivery, years	28.6 (4.7)	29.2 (3.7)	29.6 (4.6)	28.4 (4.9)	<0.001
No smoking in pregnancy, %	77.9	84.6	90.9	79.2	0.18
Parity ≥1, %	55.1	50.0	60.6	52.3	0.68
Caesarean section, %	7.6	0.3	0.7	3.1	0.88
No breastfeeding, %	25.1	15.4	27.8	24.7	0.85
Offspring					
Males, %	50.8	50.0	69.7	44.7	0.03
Birth weight (grams)	3480 (467)	3367 (649)	3889 (520)	3574 (484)	<0.001
Gestational age (weeks)	39.8 (1.3)	38.5 (1.3)	39.0 (1.3)	39.8 (1.4)	<0.001
School Entry Assessment score *N* = 6,032	13.1 (3.1)	12.7 (4.2)	13.0 (2.8)	12.9 (3.1)	0.65
Total IQ *N* = 5,282	105.3 (16.3)	103.2 (17.7)	98.7 (19.9)	103.2 (15.7)	0.09
Verbal IQ *N* = 5,307	108.2 (16.6)	108.0 (19.6)	101.0 (21.2)	106.1 (16.0)	0.07
Performance IQ *N* = 5,298	100.5 (17.0)	95.9 (14.4)	97.3 (21.0)	99.1 (16.6)	0.37
High achievement: 5+ GCSEs C-A, % *N* = 7,615	56.9	53.9	51.6	51.7	0.40
Low achievement: 0 GCSEs C-A, % *N* = 7,615	14.0	26.9	16.1	14.6	0.37

**Table 2:** Associations of preexisting diabetes, gestational diabetes, and glycosuria with School Entry Assessment score, IQ and GCSE results.

	No diabetes/glycosuria	Pre-existing diabetes	Gestational diabetes	Glycosuria
	Reference category	Mean difference (95% CI)	Mean difference (95% CI)	Mean difference (95% CI)
School Entry Assessment *N* = 6,032	*N* = 5,804	*N* = 21	*N* = 24	*N* = 183
M1	0	−0.76 (−2.05, 0.52)	−0.26 (−1.46, 0.94)	−0.55 (−0.99, −0.11)
M2	0	−0.80 (−2.03, 0.43)	−0.03 (−1.18, 1.13)	−0.36 (−0.79, 0.06)
M3 (*N* = 3,251)^‡^	0	0.04 (−1.68, 1.75)	0.30 (−1.12, 1.72)	−0.49 (−1.05, 0.08)
IQ (Total)^†^ *N* = 5,282	*N* = 5,079	*N* = 20	*N* = 23	*N* = 160
M1	0	−3.23 (−10.24, 3.78)	−6.98 (−13.52, −0.44)	−2. 16 (−4.67, 0.35)
M2	0	−2.24 (−8.82, 4.35)	−4.85 (−11.02, 1.31)	−1.47 (−3.83, 0.89)
M3 (*N* = 2,853)	0	−0.54 (−9.61, 8.52)	−5.93 (−14.24, 2.38)	−1.78 (−5.02, 1.46)
Verbal IQ^†^ *N* = 5,307	*N* = 5,102	*N* = 20	*N* = 24	*N* = 161
M1	0	−1.29 (−8.41, 5.82)	−7.87 (−14.37, −1.37)	−2.19 (−4.73, 0.35)
M2	0	−0.44 (−7.15, 6.27)	−5.95 (−12.09, 0.20)	−1.56 (−3.96, 0.84)
M3 (*N* = 2,865)^‡^	0	1.56 (−7.63, 10.74)	−9.92 (−18.34, −1.50)	−2.36 (−5.62, 0.91)
Performance IQ^†^ *N* = 5,298	*N* = 5,094	*N* = 20	*N* = 23	*N* = 161
M1	0	−5.50 (−12.89, 1.88)	−3.00 (−9.89, 3.89)	−1.52 (−4.16, 1.12)
M2	0	−4.60 (−11.76, 2.57)	−1.24 (−7.95, 5.47)	−0.90 (−3.46, 1.67)
M3 (*N* = 2,861)^‡^	0	−4.01 (−13.80, 5.78)	−0.19 (−9.17, 8.78)	−0.57 (−4.05, 2.91)

	Reference category	Odds ratio (95% CI)	Odds ratio (95% CI)	Odds ratio (95% CI)

GCSE: 5+ A*-C (high achievement) *N* = 7,615	*N* = 7,318	*N* = 26	*N* = 31	*N* = 240
M1	1	0.81 (0.37, 1.79)	0.76 (0.37, 1.56)	0.78 (0.60, 1.02)
M2	1	0.77 (0.34, 1.76)	0.84 (0.40, 1.78)	0.84 (0.64, 1.11)
M3 (*N* = 4,110)^‡^	1	0.82 (0.25, 2.71)	0.83 (0.30, 2.31)	0.92 (0.64, 1.32)
GCSE: 0 A*-C (low achievement) *N* = 7,615	*N* = 7,318	*N* = 26	*N* = 31	*N* = 240
M1	1	2.54 (1.05, 6.19)	1.23 (0.46, 3.26)	1.10 (0.76, 1.60)
M2	1	2.96 (1.17, 7.48)	1.18 (0.43, 3.23)	0.97 (0.66, 1.43)
M3 (*N* = 4,110)^‡^	1	5.06 (1.42, 18.10)	1.41 (0.38, 5.21)	1.10 (0.67, 1.79)

M1: Adjusted for sex and maternal age at birth.

M2: As in M1 plus adjustment for pre-pregnancy BMI, maternal smoking in pregnancy, parity, mode of delivery, maternal education and social class.

M3: As in M2 plus adjustment for gestational age, birth weight standardized for gestational age, and duration of breast feeding.

^†^Models for IQ-adjusted for age at assessment.

*Coefficients in the second part of the table are odds ratios (95%CI).

^‡^ *N* is reduced due to missing birth weight and breast feeding data.

**Table 3:** Associations of pre-existing diabetes, gestational diabetes, and glycosuria with School Entry Assessment score, IQ and GCSE results using imputed datasets.

	No diabetes/glycosuria	Pre-existing diabetes	Gestational diabetes	Glycosuria
	Reference category	Mean difference (95% CI)	Mean difference (95% CI)	Mean difference (95% CI)
School Entry Assessment *N* = 8,127	*N* = 7,786	*N* = 33	*N* = 39	*N* = 269
M1	0	−0.81 (−1.84, 0.24)	−0.20 (−1.18, 0.77)	−0.59(−0.97, −0.22)
M2	0	−0.97 (−1.98, 0.04)	−0.06 (−0.99, 0.86)	−0.42 (−0.77, −0.06)
M3	0	−0.93 (−1.94, 0.08)	−0.01 (−0.94, 0.91)	−0.40 (−0.76, 0.91)
IQ (Total)^†^ *N* = 6,630	*N* = 6,354	*N* = 28	*N* = 32	*N* = 216
M1	0	−5.64 (−11.57, 0.29)	−6.44 (−12.09, −0.79)	−2.69 (−4.86, −0.79)
M2	0	−4.45 (−10.04, 1.14)	−3.36 (−8.73, 2.00)	−1.98 (−4.03, 0.08)
M3	0	−4.33 (−9.94, 1.28)	−3.20 (−8.58, 2.17)	−2.00 (−4.06, 0.06)
Verbal IQ^†^ *N* = 6,630	*N* = 6,354	*N* = 28	*N* = 32	*N* = 216
M1	0	−4.10 (−10.13, 1.92)	−5.61 (−11.25, 0.04)	−3.23 (−5.43, −1.03)
M2	0	−3.07 (−8.77, 2.62)	−2.63 (−7.98, 2.72)	−2.62 (−4.71, −0.53)
M3	0	−3.08 (−8.79, 2.63)	−2.56 (−7.93, 2.80)	−2.63 (−4.72, −0.54)
Performance IQ^†^ *N* = 6,630	*N* = 6,354	*N* = 28	*N* = 32	*N* = 216
M1	0	−6.64 (−12.91, −0.36)	−5.75 (−11.99, 0.49)	−1.51 (−3.82, 0.80)
M2	0	−5.56 (−11.66, 0.53)	−3.32 (−9.42, 2.79)	−0.86 (−3.12, 1.39)
M3	0	−5.27 (−11.38, 0.85)	−3.06 (−9.17, 3.05)	−0.90 (−3.15, 1.35)

	Reference category	Odds ratio (95% CI)	Odds ratio (95% CI)	Odds ratio (95% CI)

GCSE: 5+ A*-C (high achievement) *N* = 10,198	*N* = 9,776	*N* = 38	*N* = 50	*N* = 334
M1	1	0.65 (0.34, 1.25)	0.93 (0.53, 1.66)	0.78 (0.62, 0.98)
M2	1	0.56 (0.28, 1.11)	1.11 (0.61, 2.03)	0.88 (0.69, 1.12)
M3	1	0.56 (0.28, 1.11)	1.10 (0.60, 2.02)	0.88 (0.69, 1.12)
GCSE: 0 A*-C (low achievement) *N* = 10,198	*N* = 9,776	*N* = 38	*N* = 50	*N* = 334
M1	1	1.92 (0.92, 4.03)	0.72 (0.30, 1.70)	1.31 (0.99, 1.72)
M2	1	2.48 (1.15, 5.38)	0.64 (0.26, 1.56)	1.12 (0.83, 1.50)
M3	1	2.47 (1.14, 5.35)	0.63 (0.226, 1.55)	1.12 (0.83, 1.50)

M1: Adjusted for sex and maternal age at birth.

M2: As in M1 plus adjustment for pre-pregnancy BMI, maternal smoking in pregnancy, parity, mode of delivery, maternal education and social class.

M3: As in M2 plus adjustment for gestational age, birth weight standardized for gestational age, and duration of breast feeding.

^†^Models for IQ adjusted for age at assessment.

*Coefficients in the second part of the table are odds ratios (95%CI).
